# Tissue engineering and the future of hip cartilage, labrum and ligamentum teres

**DOI:** 10.1093/jhps/hnv051

**Published:** 2015-08-11

**Authors:** Allston J. Stubbs, Elizabeth A. Howse, Sandeep Mannava

**Affiliations:** 1. Division of Sports Medicine, Department of Orthopaedic Surgery, Wake Forest University School of Medicine, Medical Center Boulevard, Winston-Salem, North Carolina 27157, USA; 2. Department of Emergency Medicine, Kaiser Permanente Walnut Creek Medical Center, 1425 S. Main St, Walnut Creek, CA, 94596, USA

## Abstract

As the field of hip arthroscopy continues to evolve, the biological understanding of orthopaedic tissues, namely articular cartilage, labral fibro-cartilage and the ligamentum teres continues to expand. Similarly, the need for biological solutions for the pre-arthritic and early arthritic hip continues to be a challenge for the sports medicine surgeon and hip arthroscopist. This article outlines existing biological and tissue-engineering technologies, some being used in clinical practice and other technologies being developed, and how these biological and tissue-engineering principals may one day influence the practice of hip arthroscopy. This review of hip literature is specific to emerging biological technologies for the treatment of chondral defects, labral tears and ligamentum teres deficiency. Of note, not all of the technologies described in this article have been approved by the United States Food and Drug Administration and some of the described uses of the approved technologies should be considered ‘off-label’ uses.

## INTRODUCTION

Hip arthroscopy has evolved significantly since Burman’s [1] first 1931 report of the arthroscopic appearance of intra-articular structures. As hip arthroscopy has rapidly evolved, the specific instrumentation for performing the operations as well as the indications for surgery has expanded [2]. As the field of hip arthroscopy continues to evolve, the biological understanding of orthopaedic tissues, namely articular cartilage, labral fibro-cartilage and the ligamentum teres, continues to expand. Similarly, the need for biological solutions for the pre-arthritic and early arthritic hip continues to be a challenge for the sports medicine surgeon and hip arthroscopist.

This article will briefly review the native hip anatomy and common pathology encountered in patients undergoing hip arthroscopy. The focus will then shift to outlining existing biological and tissue engineer technologies, some being used in clinical practice and other technologies being developed, and how these biological and tissue-engineering principals may one day influence the practice of hip arthroscopy. This article is a forward thinking and certainly not all inclusive, as many of the technologies described have not been specifically tested in the hip joint; however, after review of the article, the reader will have a better understanding of recent tissue-engineering and biological technologies that may influence clinical practice in the years to come. We conclude this article by briefly mentioning some of the techniques used at our institutes’ regenerative medicine laboratory, although not all of the techniques described are being investigated for orthopaedic purposes, we do hope the mention of these technologies will result in future orthopaedic and musculoskeletal investigation. Of note, some of the described uses of the approved technologies should be considered ‘off-label’ uses as not all of the technologies described in this article have been approved by the United States Food and Drug Administration to be used as described.

## NATIVE HIP ANATOMY AND PATHOLOGY

The hip is a weight-bearing ball and socket joint that is deeply seated and congruent, with a great deal of anatomical constraint, especially when compared with the shoulder joint. Two main forms of cartilage exist in the hip joint, articular hyaline cartilage, from innominate fusion of osteochondral complexes, and labral fibrocartilage [3–5]. The hip joint also contains the ligamentum teres, which does have an embryological and early developmental role in hip joint formation, yet the role in pathology remains of some debate. The ligamentum teres may not simply represent vestigial anatomy, with some recent literature suggesting a more structural role [6–8]. The hip joint derives its vascularity from innominate fusion and much of the innervation of the joint is shared from surrounding layers and muscle-tendon units [5].

The challenge for the hip arthroscopist is to address various lesions of the articular cartilage, fibrocartilagenous labrum and the ligamentum teres in a minimally invasive manner. The pre-arthritic and early arthritic hip can be particularly challenging surgically because of its deep anatomic location, and the relatively high physiological loads, forces and stresses seen by the joint.

## HIP ARTICULAR CARTILAGE

For the hip arthroscopist, articular cartilage defects can be addressed in the pre-arthritic or early arthritic stage. The gold standard for end-stage hip arthritis is total hip arthroplasty; but there are many patients who have articular cartilage wear that may not be significant enough to warrant total joint arthroplasty [9–11]. To date, there are several articular cartilage strategies that have been employed to help restore focal and larger cartilage defects in the active patient. Some of these articular cartilage strategies include autologous chondrocyte implantation , microfracture, composite grafting [e.g. synthetic TruFit bone graft substitute (Smith & Nephew Inc., Andover, MA, USA)], osteochondral autograft transfer system (Arthrex Inc., Naples, FL, USA) and fresh frozen allograft [12–20].

When examining cartilage, the ‘gold-standard’ is hyaline cartilage, which is the native cartilage of the hip joint; mainly comprised of type II collagen and layers of functional cells and extracellular matrix [12, 21, 22]. Marrow stimulation or microfracture technique has been utilized for hip articular cartilage restoration, with the goal of stimulating pluripotent cells of the inner pelvic table and proximal femur to restore focal areas of cartilage defects [15–18, 21, 22]. The microfractured cartilage that replaces the injured region of articular cartilage does share some characteristics of hyaline cartilage; however, there are key matrix components, including aggregans that are not expressed optimally in the regenerated cartilage [21].

There are numerous reports in the literature that demonstrate favourable clinical outcomes when performing microfracture for the articular surface of the hip joint [15–18]. Philippon *et al.* [15] examined the percentage of filled articular cartilage defect on the acetabular side of the hip joint after microfracture surgery as evaluated by second-look revision hip arthroscopy. They report excellent results of 95–100% coverage of the isolated acetabular chondral lesions at an average of 20 months follow-up for eight of their nine patients [15]. Similarly, Karthikeyan *et al.* [16] demonstrated adequate macroscopic fill of acetabular articular defects with associated femoral acetabular impingement on second-look arthroscopy after microfracture was performed an average of 17 months prior. In addition, microscopic histological evaluation of the tissue demonstrated fibrocartilage in the region of the microfracture. In all, 19 of the 20 patients in the series demonstrated mean fill of 96%. Domb *et al.* [17] studied patient reported outcome measures and demonstrated significant clinical improvement after microfracture was performed in their patient cohort at 2-year follow up. The outcome measures assessed were the modified Harris Hip Sore, the Non-Arthritic Hip Score (NAHS), the Hip Outcome Score–Activities of Daily Living, and the Hip Outcome Score—Sport Specific Subscale. These scores improved in their cohort; interestingly the improvement occurred for both a workers’ compensation and a non-workers’ compensation cohort at 2 year follow up when compared with pre-operative scores [17]. In elite athletes who underwent hip arthroscopy with microfracture compared with elite athletes who underwent hip arthroscopy without microfracture, McDonald *et al.* [18] demonstrated that the additional procedure of microfracture surgery did not preclude the athlete from returning to a high level of competition.

Other, more isolated case series have demonstrated some early success with mosaicplasty and autologous chondrocyte transplantation for the treatment of hip articular defects [19, 20]. Hart *et al.* [19] demonstrated the feasibility of mosaicplasty of femoral head cartilage defects with autologous grafting from non-weight bearing portions of the knee in their case report. The authors of this report state that their experiences with mosaicplasty techniques in the hip are limited but theoretically, in certain clinical situations, may benefit the patient with a cartilage defect. Fontana *et al.* [20] compared the results of autologous chondrocyte transplantation (*n* = 15 patients) versus simple debridement (*n* = 15 patients) for hip acetabular chondral defects of grade three or four Outerbridge classification, with more than 2 cm^2^ area. They demonstrated an improved Harris Hip Score at a mean of 74 months after the procedure in the autologous chondrocyte transplantation group when compared with the simple debridement group. As a whole, early isolated case series and reports have demonstrated feasibility and some favourable outcomes for the arthroscopic treatment of hip chondral lesions [15–22].

## HIP LABRUM

The hip labrum is a unique fibrocartilagenous biological structure. It has multiple purposes including increasing joint stability, congruity and overall depth of the ball-and-socket joint, allowing for a biological seal that is thought to be protective to the hip articular cartilage [5, 23, 24]. In some instances of injury, primary repair of the hip labrum is sufficient with suture anchor constructs in order to reconstitute the mechanical function of the labrum. In circumstances of more severe injury, hip labral reconstruction must be undertaken in order to re-constitute the labrum’s function [25–29]. Graft choices’ reports in the literature include both allograft and autograft from gracilis/hamstring, quadriceps tendon, hamstring, iliotibial band, ligamentum teres or tensor fascia lata [25–29].

Costa Rocha *et al.* [26] reported a case series of four patients followed for 2 years who underwent labral reconstruction with hamstring semi-tendinous allograft, with improved functions in three of their four patients as demonstrated by an improved Oxford hip score, HOS and Global Treatment Outcome Score. Park and Ko [27] published a case report demonstrating the feasibility of using quadriceps tendon as an autograft option for labral reconstruction, although their follow up at the time of publication was only 3 months. Domb *et al.* [28] report a cohort study of 11 labral reconstruction versus 22 labral resection patients with significant improvement in the NAHS and hip outcome score for activities of daily living for the reconstruction cohort at a minimum of 2 years of follow-up. Ayeni *et al.* [25] systematically reviewed the available literature regarding hip labral reconstruction in 2014 and concluded that there are promising short-term functional and patient-reported outcomes benefits to reconstruction. Although, they did note that long-term follow-up for patients was not reported in the literature [25].

Potential graft choices for reconstruction can include autograft, allograft, xenograft or biological tissue-engineered scaffolds, each with their own benefits and shortcomings. Autografts allow for the labrum to be reconstructed with a patient’s own tissue, but harvest of the graft may lead to donor site morbidity, which in some instances can be severe. Allografts offer an off-the-shelf solution for labral reconstruction but carry a small risk of rejection and disease transmission. Both autograft and allograft technologies undergo a period of ‘labralization’ in which there is local inflammation, followed by remodelling whereby the reconstructed graft tissue undergoes biological transformation into labral tissue [30–32]. A tissue engineered xenograft solution or scaffold solution may, in theory, improve integration and speed ‘labralization’ of the tissue by decreasing the time needed for remodelling. In theory, an ideal tissue-engineered solution for labral reconstruction would be naturally derived, compatible with the host tissue and immune system, have refined architecture to allow for rapid integration, and have sufficient biomechanical integrity to withstand post-surgical rehabilitation [30–32]. Although large series with long-term clinical follow-up of labral reconstruction with tissue engineered graft solutions are not presently available, the field of tissue engineering offers an exciting potential solution that could theoretically improve recovery time and post-operative morbidity, along with optimizing ultimate integration and function after labral reconstruction surgery.

## LIGAMENTUM TERES

Recently, the biomechanical role of the ligamentum teres has been better appreciated and defined [6–8]. In certain instances, significant ligamentum teres disease can be associated with pain and general hip dysfunction in the pre-arthritic hip. The biomechnical role of the ligamentum teres in various hip positions has been studied in an animal model system [6]. In fact, isocentric reattachment of the ligamentum teres is not only feasible in an animal model system, but it was demonstrated to serve as a ‘natural check-rein’ to dislocation of the hip joint. Further, the ligamentum teres reconstruction in an animal model system was successfully performed without restricting hip motion or causing abnormal cartilage pressures [6]. Similar techniques have been described in an isolated case report by Simpson *et al.* [8], in which the ligamentum teres reconstruction was performed for recurrent pain associated with feelings of hip instability despite having undergone previous hip arthroscopy. In theory, graft options such as those available for anterior cruciate ligament of the knee reconstruction would be feasible options in the future. The use of both allografts and autografts is plausible, and both the advantages and disadvantages have been reviewed previously in this article. Similarly, future work may focus on an off-the-shelf tissue engineered solution or graft option for ligamentum teres reconstruction, using principals currently being applied for biological engineering of orthopaedic tissue as previously described [32].

## EMERGING TISSUE-ENGINEERING TECHNOLOGIES

This section provides the general framework of the emerging biological field of regenerative medicine. In all, there has been a recent effort at many institutions, including our own, to combine diverse expertise to address various maladies in modern medicine. Although in its infancy, regenerative medicine is a field that has some exciting potential for orthopaedic applications, specifically for hip arthroscopy. Currently, specific efforts for utilizing and applying regenerative medicine technologies for hip arthroscopy purposes are limited. However, by providing a brief discussion, we hope to highlight how this field may potentially inspire future solutions to many of the shortcomings of biological healing and reconstruction that currently limit hip arthroscopy [33–39].

Regenerative medicine is a relatively new and emerging field in which many medical, biological and physical science principals are being applied to address the shortage of biological organs and tissues available for transplantation or therapy, secondary to primary organ or tissue pathology and failure. The field of regenerative medicine is more diverse than simply attempting to grow replacement organs. The field as a whole can be broadly divided into tissue engineering, diagnostic platforms, cellular therapies, healing therapies and supporting technologies. Tissue engineering is a branch of regenerative medicine in which replacement tissue and organs, as well as other body parts like skin or ears are being developed *ex vivo*. Diagnostic platforms have also emerged from regenerative medical experimentation, where genetic and pathogen detection tests have been developed, and engineered tissue is being used for pre-clinical drug testing. Cellular therapies are constantly being explored, where pluripotent cells, such as stem cells, are being investigated for their reparative properties in the setting of pathological states. Further, healing therapies, which may change the diseased environment, are being actively investigated in regenerative medicine. An example of healing therapies would be a biological dressing used to treat a contaminated wound or improve healing after a burn by modifying the injured environment. Finally, supporting technologies have emerged as an essential aspect of regenerative medicine, where cell harvesting kits and novel delivery techniques have been developed as part of the regenerative medicine armamentarium to be used as tools for implementing regenerative technology in the clinical setting [36–40].

**Fig. 1.**
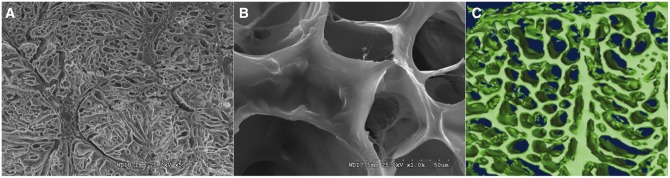
Images of a tissue-engineered pig xenograft, naturally derived scaffold for tendon, ligament or labral reconstruction. **A** Represents an overview scanning electron microscopy view of the macrostructure of the tissue-engineered graft. The pore-size of the xenograft has been optimized for cell infiltration while still providing mechanical strength. **B** Shows a closer view of pore architecture of the xenograft. **C** A three-dimensional microcomputer tomography reconstruction of the pore architecture of the xenograft. Three-dimensional reconstruction can be used to study microporosity and interconnectedness of the graft architecture (special thanks to Drs Patrick W. Whitlock and Thorsten M. Seyler for providing the xenograft samples depicted in this image).

**Fig. 2.**
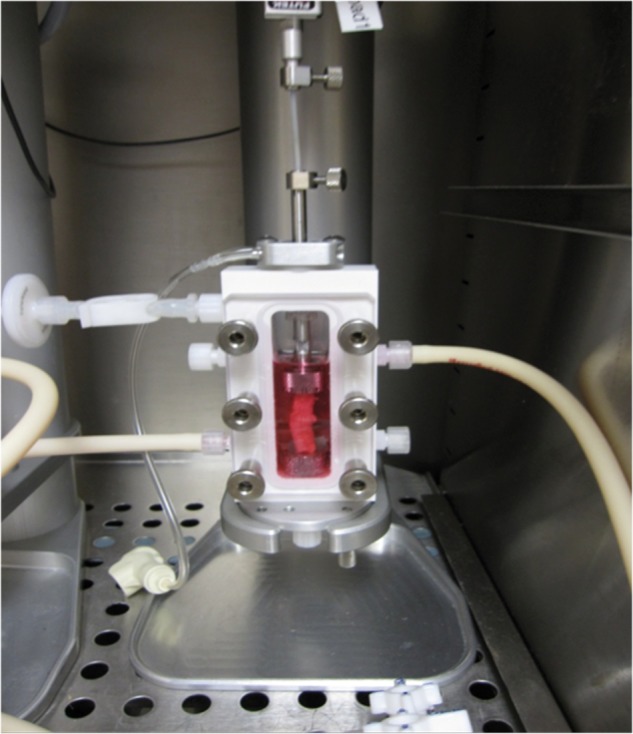
A commercially available bioreactor is depicted in this image (DynaGen bioreactor system, Tissue Growth Technologies, Minnetonka, MN, USA). A tissue-engineered graft can be seeded with pluripotent cells and then be maintained in culture media while having mechanical stimulus applied to the graft in order to aid in differentiation and maturation of the tissue-engineered construct.

**Fig. 3.**
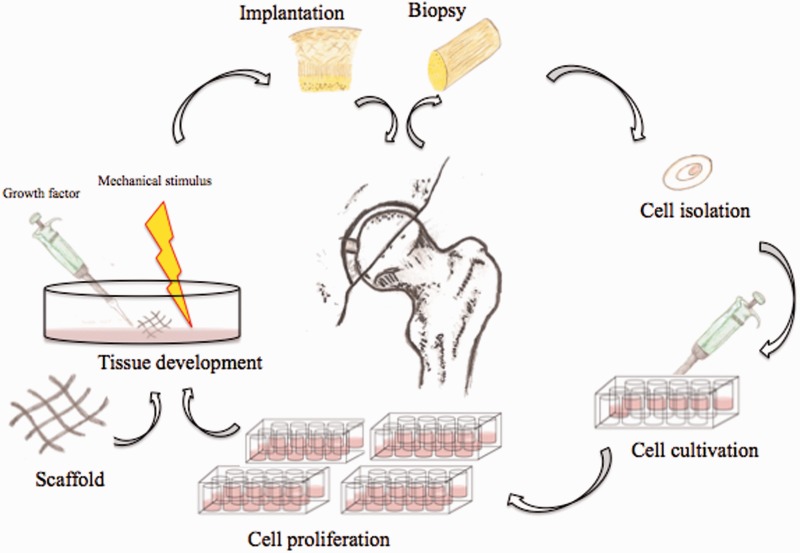
Schematic diagram demonstrating an artist rendition of the tissue-engineering pathway of harvesting pluripotent stem cells, culturing the cells *in vitro*, differentiation of the cells and potential re-implantation for the purposes of hip preservation surgery via arthroscopic techniques.

**Fig. 4.**
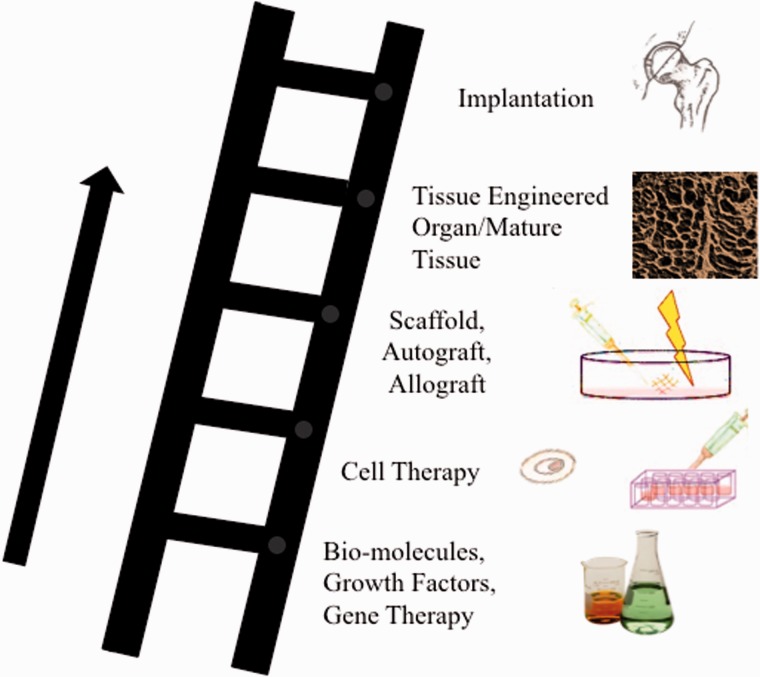
Schematic diagram demonstrating the concept of the reconstructive ladder of tissue-engineering and regenerative medicine. As one proceeds up the rungs of the reconstructive ladder, the regenerative medicine construct becomes more complex and structured. Based upon the clinical scenario, one can easily attain a tissue-engineered solution from any rung of reconstructive ladder.

An example of regenerative medicine technology would be harvesting and then isolating pluripotent stems cells from a patient. Those cells could then be cultured *in vitro* so that the cells continue to proliferate. These proliferative cells could then be seeded on a scaffold (Fig. 1), which allow for three-dimensional orientation and potentially will help with organization and differentiation of the stems cells. Various growth factors and mechanical stimuli, such as those provided by a bioreactor (Fig. 2), can be used to help in the stem cell differentiation and remodelling into mature, end-organ, tissue. At the end of the tissue-engineering process, the surgeon may have an engineered tissue for replacement and reconstruction, which in theory can be optimized to speed the healing and recovery process (Fig. 3) [36–39]. When faced with a clinical dilemma in which a tissue-engineered solution may be of value, the surgeon can think of varying degrees of regenerative medicine complexity based upon the theory of the ‘reconstructive ladder’ (Fig. 4). As one moves up the rungs of the reconstructive ladder, the complexity of the regenerative medicine solution increases. Lower rungs consist of molecular or cell-based therapies that may aide in producing a more conducive healing environment. Full organ transplantation or use of a mature tissue-engineered construct consisting of a naturally derived scaffold that has been seeded with stem cells and matured on a bioreactor is a more complex and ‘higher rung’ solution on the reconstructive ladder.

Our institution has completed the tissue engineering of urethra, vagina, bladder neck, ureter and bladder. Currently, blood vessel, heart valve, sphincter muscle, ear, finger/digits, kidney, nerve, skin and skeletal muscle are being developed [35–39]. The use of the novel technology of bioprinting or micro-organoids have allowed for the production of the microscopic liver structure, bladder tissue structure, testis, cardiac muscle and kidney structure [35]. It is the hope that with continued collaborative efforts, that these technologies may be applied to orthopaedic sports medicine and arthroscopic applications.

## CONCLUSION AND DISCUSSION OF FUTURE DIRECTIONS

The future of hip arthroscopy and the use of biological agents for arthroscopic hip surgery are exciting. The primary limitations are mainly cell and mechanically based. The use of tissue engineering, as well as regenerative medicine technology does represent an exciting paradigm for addressing orthopaedic sports medicine problems. Although biological and tissue engineering solutions for hip arthroscopy are presently limited, the future has much potential for the continued evolution of existing technologies that may ultimately improve surgical outcomes, speed recovery and decrease post-operative rehabilitation limitations.

### CONFLICT OF INTEREST STATEMENT

Allston J Stubbs serves as a paid consultant for a company or supplier: Smith and Nephew; he or a direct family member has stock or stock options in Johnson and Johnson; received research support from a company or supplier as PI Bauerfeind AG; he serves on editorial boards of VuMedi.com and Journal of Arthroscopy; he has board membership and/or committee appointments for the following organizations: International Society for Hip Arthroscopy, American Orthopaedic Society for Sports Medicine, Arthroscopy Association of North America.

Sandeep Mannava has received institutional research support from Wake Forest Innovations for work not related to the subject of this publication and he has been issued a United States patent entitle ‘tissue tensioning deviced and related methods.’

Elizabeth A Howse has no potential conflict of interest to declare.

## References

[1] Burman MS Arthroscopy or the direct visualization of joints: an experimental cadaver study. 1931. Clin Orthop Relat Res 2001; 390:5–9. 11550876 10.1097/00003086-200109000-00003

[2] Colvin AC Harrast J Harner C Trends in hip arthroscopy. J Bone Joint Surg Am 2012; 94:e23. 22336982 10.2106/JBJS.J.01886

[3] Bharam S Labral tears, extra-articular injuries, and hip arthroscopy in the athlete. Clin Sports Med 2006; 25:279–92. 16638491 10.1016/j.csm.2006.01.003

[4] Groh MM Herrera J A comprehensive review of hip labral tears. Curr Rev Musculoskelet Med 2009; 2:105–17. 19468871 10.1007/s12178-009-9052-9 PMC2697339

[5] Kalhor M Horowitz K Beck M Vascular supply to the acetabular labrum. J Bone Joint Surg Am 2010; 92: 2570–5. 21048175 10.2106/JBJS.I.01719

[6] Hosalkar HS Varley ES Glaser D Isocentric reattachment of ligamentum teres: a porcine study. J Pediatr Orthop 2011; 31: 847–52. 22101662 10.1097/BPO.0b013e31822e0276

[7] de Sa D Phillips M Philippon MJ Ligamentum teres injuries of the hip: a systematic review examining surgical indications, treatment options, and outcomes. Arthroscopy 2014; 30:1634–41. 25125381 10.1016/j.arthro.2014.06.007

[8] Simpson JM Field RE Villar RN Arthroscopic reconstruction of the ligamentum teres. Arthroscopy 2011; 27: 436–41. 21292435 10.1016/j.arthro.2010.09.016

[9] Domb BG Gui C Lodhia P How much arthritis is too much for hip arthroscopy: a systematic review. Arthroscopy 2015; 31: 520–9. 25543247 10.1016/j.arthro.2014.11.008

[10] Philippon MJ Briggs KK Carlisle JC Joint space predicts THA after hip arthroscopy in patients 50 years and older. Clin Orthop Relat Res 2013; 471:2492–6. 23292888 10.1007/s11999-012-2779-4 PMC3705033

[11] Philippon MJ Schroder ESBG Briggs KK Hip arthroscopy for femoroacetabular impingement in patients aged 50 years or older. Arthroscopy 2012; 28: 59–65. 21982390 10.1016/j.arthro.2011.07.004

[12] Lewis PB McCarty LP 3rd Kang RW Basic science and treatment options for articular cartilage injuries. J Orthop Sports Phys Ther 2006; 36: 717–27. 17063834 10.2519/jospt.2006.2175

[13] Farr J Cole B Dhawan A Clinical cartilage restoration: evolution and overview. Clin Orthop Relat Res 2011; 469:2696–705. 21240578 10.1007/s11999-010-1764-z PMC3171560

[14] Cole BJ Pascual-Garrido C Grumet RC Surgical management of articular cartilage defects in the knee. Instr Course Lect 2010; 59:181–204. 20415379

[15] Philippon MJ Schenker ML Briggs KK Can microfracture produce repair tissue in acetabular chondral defects? Arthroscopy 2008; 24:46–50. 18182201 10.1016/j.arthro.2007.07.027

[16] Karthikeyan S Roberts S Griffin D Microfracture for acetabular chondral defects in patients with femoroacetabular impingement: results at second-look arthroscopic surgery. Am J Sports Med 2012; 40:2725–30. 23136178 10.1177/0363546512465400

[17] Domb BG El Bitar YF Lindner D Arthroscopic hip surgery with a microfracture procedure of the hip: clinical outcomes with two-year follow-up. Hip Int 2014; 24:448–56. 25096453 10.5301/hipint.5000144

[18] McDonald JE Herzog MM Philippon MJ Return to play after hip arthroscopy with microfracture in elite athletes. Arthroscopy 2013; 29:330–5. 23290181 10.1016/j.arthro.2012.08.028

[19] Hart R Janecek M Visna P Mosaicplasty for the treatment of femoral head defect after incorrect resorbable screw insertion. Arthroscopy 2003; 19:E1–5. 14673462 10.1016/j.arthro.2003.10.025

[20] Fontana A Bistolfi A Crova M Arthroscopic treatment of hip chondral defects: autologous chondrocyte transplantation versus simple debridement—a pilot study. Arthroscopy 2012; 28:322–9. 22142720 10.1016/j.arthro.2011.08.304

[21] Frisbie DD Oxford JT Southwood L Early events in cartilage repair after subchondral bone microfracture. Clin Orthop Relat Res 2003; 407:215–27. 12567150 10.1097/00003086-200302000-00031

[22] Frisbie DD Morisset S Ho CP Effects of calcified cartilage on healing of chondral defects treated with microfracture in horses. Am J Sports Med 2006; 34:1824–31. 16832126 10.1177/0363546506289882

[23] Ferguson SJ Bryant JT Ganz R The influence of the acetabular labrum on hip joint cartilage consolidation: a poroelastic finite element model. J Biomech 2000; 33:953–60. 10828325 10.1016/s0021-9290(00)00042-7

[24] Ferguson SJ Bryant JT Ganz R The acetabular labrum seal: a poroelastic finite element model. Clin Biomech (Bristol, Avon) 2000; 15:463–8. 10.1016/s0268-0033(99)00099-6 10771126

[25] Ayeni OR Alradwan H de Sa D The hip labrum reconstruction: indications and outcomes—a systematic review. Knee Surg Sports Traumatol Arthrosc 2014; 22(4):737–43. 24318405 10.1007/s00167-013-2804-5

[26] Costa Rocha P Klingenstein G Ganz R Circumferential reconstruction of severe acetabular labral damage using hamstring allograft: surgical technique and case series. Hip Int 2013; 23(Suppl 9):S42–53. 24318364 10.5301/HIP.2013.11662

[27] Park SE Ko Y Use of the quadriceps tendon in arthroscopic acetabular labral reconstruction: potential and benefits as an autograft option. Arthrosc Tech 2013; 2:e217–9. 24265987 10.1016/j.eats.2013.02.003 PMC3834660

[28] Domb BG El Bitar YF Stake CE Arthroscopic labral reconstruction is superior to segmental resection for irreparable labral tears in the hip: a matched-pair controlled study with minimum 2-year follow-up. Am J Sports Med 2014; 42:122–30. 24186974 10.1177/0363546513508256

[29] Matsuda DK Burchette RJ Arthroscopic hip labral reconstruction with a gracilis autograft versus labral refixation: 2-year minimum outcomes. Am J Sports Med 2013; 41:980–7. 23548806 10.1177/0363546513482884

[30] Whitlock PW Smith TL Poehling GG A naturally derived, cytocompatible, and architecturally optimized scaffold for tendon and ligament regeneration. Biomaterials 2007; 28:4321–9. 17610948 10.1016/j.biomaterials.2007.05.029

[31] Whitlock PW Seyler TM Parks GD A novel process for optimizing musculoskeletal allograft tissue to improve safety, ultrastructural properties, and cell infiltration. J Bone Joint Surg Am 2012; 94:1458–67. 22786867 10.2106/JBJS.K.01397

[32] Whitlock P Seyler T Mannava S A tissue-engineered approach to tendon and ligament reconstruction. In: Doral MN (ed). Sports Injuries. Berlin: Springer, 2012, 1185–91.

[33] Hunsberger J Harrysson O Shirwaiker R Manufacturing road map for tissue engineering and regenerative medicine technologies. Stem Cells Transl Med 2015; 4:130–5. 25575525 10.5966/sctm.2014-0254 PMC4303363

[34] Bertram TA Tentoff E Johnson PC Hurdles in tissue engineering/regenerative medicine product commercialization: a pilot survey of governmental funding agencies and the financial industry. Tissue Eng A 2012; 18:2187–94. 10.1089/ten.TEA.2012.0186 22838399

[35] Sadri-Ardekani H Atala A Regenerative medicine for the treatment of reproductive system disorders: current and potential options. Adv Drug Deliv Rev 2015; 82–83:145–52 10.1016/j.addr.2014.10.019 25453265

[36] Olson JL Atala A Yoo JJ Tissue engineering: current strategies and future directions. Chonnam Med J 2011; 47:1–13. 22111050 10.4068/cmj.2011.47.1.1 PMC3214857

[37] Atala A Regenerative medicine strategies. J Pediatr Surg 2012; 47:17–28. 22244387 10.1016/j.jpedsurg.2011.10.013

[38] Atala A Engineering organs. Curr Opin Biotechnol 2009; 20:575–92. 19896823 10.1016/j.copbio.2009.10.003

[39] Atala A Engineering tissues, organs and cells. J Tissue Eng Regen Med 2007; 1:83–96. 18038397 10.1002/term.18

[40] Poranki D Whitener W Howse S Evaluation of skin regeneration after burns in vivo and rescue of cells after thermal stress in vitro following treatment with a keratin biomaterial. J Biomater Appl 2013; 29:26–35. 24272161 10.1177/0885328213513310

